# Redefining Endometrial Decidualization: The Central Role of the ER Stress–Immune–Metabolic Axis

**DOI:** 10.3390/ijms27104382

**Published:** 2026-05-14

**Authors:** Özdem Karaoğlan, Özgül Tap, İbrahim Ferhat Ürünsak

**Affiliations:** 1 Department of Histology and Embryology, Faculty of Medicine, Cukurova University, 01330 Adana, Turkey; otap@cu.edu.tr; 2 Department of Obstetrics and Gynecology, Faculty of Medicine, Cukurova University, 01330 Adana, Turkey; iurunsak@cu.edu.tr

**Keywords:** endometrial decidualization, endoplasmic reticulum stress, unfolded protein response, immune regulation, metabolic reprogramming

## Abstract

Decidualization in the human endometrium is not merely a hormone-dependent differentiation process; rather, it represents a multilayered adaptive program characterized by the tight integration of immune regulation, metabolic reprogramming, and cellular stress responses. In this review, endoplasmic reticulum (ER) stress and the associated unfolded protein response (UPR) are proposed as central regulatory mechanisms governing this process. Triggered by increased protein synthesis and secretory demand, UPR activation under physiological conditions preserves proteostasis and supports the secretory capacity of stromal cells. In contrast, chronic or dysregulated activation leads to a maladaptive response characterized by apoptosis, inflammation, and metabolic dysfunction. UPR signaling pathways shape immune tolerance through their effects on macrophage polarization, uterine natural killer (uNK) cell function, and T cell balance. At the metabolic level, adenosine monophosphate-activated protein kinase (AMPK) regulates cellular adaptation through bidirectional interactions with mitochondrial function and redox homeostasis. Within this framework, the ER stress–immune–metabolic axis operates not as a linear pathway but as a dynamic network incorporating multiple feedback loops, thereby constituting a critical threshold mechanism that determines the success of decidualization. Disruption of this axis provides a shared mechanistic basis for pathologies such as recurrent implantation failure, pregnancy loss, and preeclampsia. From a therapeutic perspective, agents including chemical chaperones, UPR modulators, AMPK activators, and anti-inflammatory compounds hold translational potential by targeting these pathological feedback circuits. However, key knowledge gaps remain, particularly regarding the cell type-specific and temporal regulation of ER stress, the molecular boundaries defining the transition from adaptive to pathological states, and interspecies differences. Future studies employing single-cell omics approaches and functional in vivo models will be essential to elucidate the dynamic organization of this axis and to enable the development of targeted and personalized therapeutic strategies.

## 1. Introduction

The human endometrium is a highly dynamic tissue with remarkable functional plasticity, undergoing continuous cyclical changes under the regulation of estrogen and progesterone throughout reproductive life [1,2,3]. Transitions between the proliferative, secretory, and menstrual phases of the menstrual cycle are not limited to morphological alterations but also involve extensive transcriptional, immunological, and metabolic reprogramming [2,4]. In particular, decidualization initiated under progesterone influence is characterized by the transformation of stromal cells from a fibroblast-like phenotype into highly secretory decidual cells, with prolactin and insulin-like growth factor-binding protein-1 (IGFBP-1) serving as key markers of this differentiation process [1,5]. Recent molecular and single-cell analyses indicate that decidualization is not solely a hormone-driven response but rather a multilayered adaptive program integrating immune cell–stromal cell interactions, energy metabolism, and extracellular matrix (ECM) remodeling [4,6,7,8]. Within this context, ER stress and the associated UPR emerge not merely as passive consequences of cellular stress but as dynamic regulatory platforms central to this adaptive process [9,10,11]. Increased protein synthesis and secretory demand impose substantial stress on the ER, leading to activation of the three principal UPR branches: protein kinase RNA-like ER kinase (PERK), inositol-requiring enzyme 1 alpha (IRE1α), and activating transcription factor 6 (ATF6) [9,10,12]. Initially, this activation elicits an adaptive response that enhances protein-folding capacity and balances translational load [9,11]. However, UPR signaling extends beyond the maintenance of proteostasis, exerting direct effects on immune modulation and metabolic pathway reprogramming [10,11]. The IRE1α–XBP1 axis regulates secretory cell function and cytokine production, while the PERK–ATF4 pathway interacts with redox homeostasis and energy metabolism; ATF6, in turn, plays a critical role in sustaining secretory capacity [9,12]. From an immunological perspective, ER stress represents a key regulatory node governing the balance between inflammation and tolerance within the decidual microenvironment [6,7,10]. UPR activation influences macrophage polarization, uNK cell function, and the T cell subset equilibrium [13,14,15].

Adaptive ER stress has been associated with regulatory T cell (Treg) stability and anti-inflammatory cytokine production, thereby supporting trophoblast tolerance [10,11]. In contrast, chronic or excessive ER stress may promote CHOP-mediated apoptosis and NF-κB activation, amplifying inflammatory responses and contributing to the breakdown of immune tolerance and implantation failure [9,10,16]. From a metabolic standpoint, decidualization requires extensive reprogramming, including alterations in glucose metabolism, lipid biosynthesis, and mitochondrial remodeling [17,18]. ER stress modulates these processes through energy-sensing pathways such as AMPK and mechanistic target of rapamycin (mTOR), thereby enabling adaptation to cellular energy status [19,20,21]. AMPK activation exerts a protective role against ER stress by reducing the protein-folding burden, optimizing mitochondrial function, and limiting oxidative stress [19,20]. However, disruption of this balance leads to increased reactive oxygen species production and energy insufficiency, accelerating the transition of the UPR toward a maladaptive state and exacerbating cellular damage [21,22].

Within this framework, we propose the central hypothesis that UPR activation triggered by ER stress during decidualization constitutes a higher-order regulatory network that coordinately governs immune tolerance and metabolic adaptation, functioning as an integrated “ER stress–immune–metabolic axis”. While balanced activation of this axis is essential for successful decidualization and implantation, its chronic or dysregulated activation is associated with increased inflammation, loss of immune tolerance, and metabolic dysfunction, contributing to pathologies such as recurrent implantation failure and pregnancy loss [13,14,16,22].

## 2. Decidualization: An Integrated Biological System

Decidualization extends beyond a classical hormone-dependent cellular differentiation process and represents a multidimensional adaptive program in which structural, immune, and metabolic components are coordinately reprogrammed [3,4,5]. During this process, stromal cells acquire a secretory phenotype, while the ECM undergoes extensive remodeling, intercellular communication is enhanced, and a functional interface between the embryo and endometrium is established [23,24,25,26]. The increased energetic demand necessitates metabolic reprogramming, characterized by the regulation of glucose uptake, glycolytic flux, and mitochondrial function, alongside concurrent modulation of redox balance and cellular stress responses [17,18,27,28]. Rather than being suppressed, the immune system is dynamically restructured to establish a finely tuned balance between inflammation and tolerance. In this context, uNK cells, macrophages, and other innate and adaptive immune cell subsets actively contribute to the functional reorganization of the decidual microenvironment [5,14,29]. This multilayered adaptation imposes substantial stress on the endoplasmic reticulum due to increased protein synthesis and secretory demand, thereby triggering a physiological ER stress response [9,12,16].

## 3. Endoplasmic Reticulum Stress and the Unfolded Protein Response: A Central Regulator of Decidualization

**Figure 1:**
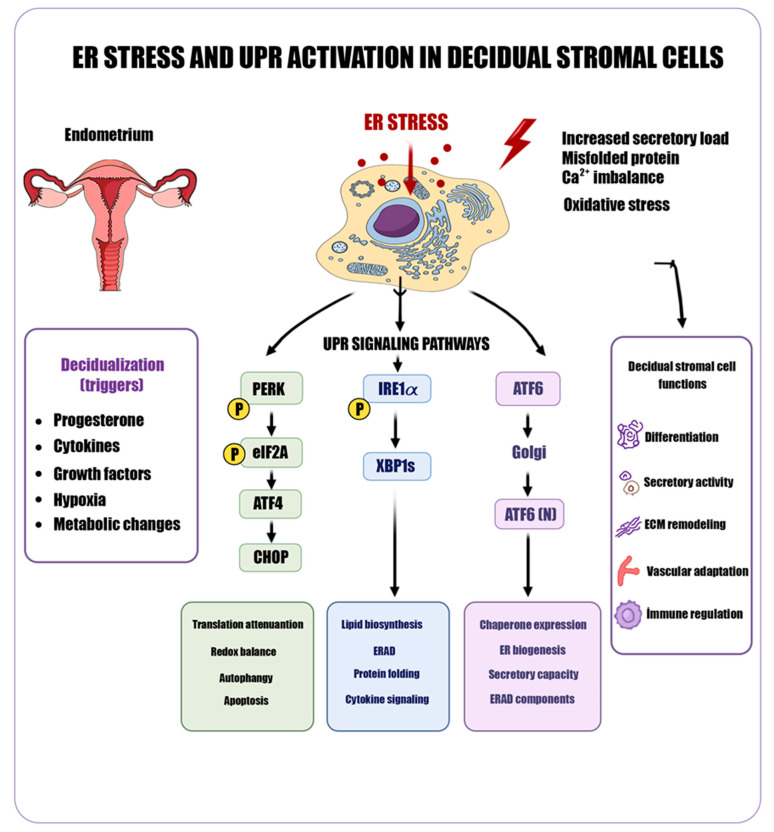
ER stress and UPR activation in decidual stromal cells. Decidualization-related stimuli (progesterone, cytokines, growth factors, hypoxia, and metabolic changes) increase secretory load, misfolded protein accumulation, Ca^2+^ imbalance, and oxidative stress, leading to ER stress. This activates the three UPR branches: PERK (eIF2α–ATF4–CHOP), IRE1α (XBP1s), and ATF6 (ATF6(N)). These pathways are associated with translational control, protein folding/ERAD, lipid biosynthesis, and stress responses, collectively supporting decidual stromal cell functions such as differentiation, secretion, extracellular matrix remodeling, vascular adaptation, and immune regulation.

Decidualization is characterized by increased protein synthesis and secretory activity, imposing a substantial burden on the ER and thereby triggering activation of the UPR [9,12,16]. This response is coordinated through three principal sensors: PERK, IRE1α, and ATF6 [9,10]. Upon PERK activation, eukaryotic initiation factor 2 alpha (eIF2α) is phosphorylated, leading to a transient attenuation of global protein translation and a consequent reduction in the influx of newly synthesized proteins into the ER. In parallel, translation of activating transcription factor 4 (ATF4) is selectively enhanced, promoting the expression of genes involved in amino acid metabolism, redox homeostasis, and cellular stress adaptation [9]. The IRE1α branch, through its endoribonuclease activity, mediates the unconventional splicing of X-box binding protein 1 (XBP1) mRNA, generating the active XBP1s form. XBP1s upregulates ER chaperones (such as BiP/GRP78), protein-folding enzymes, and components of the ER-associated degradation (ERAD) pathway, while also supporting ER membrane biogenesis and organelle expansion [10,12]. ATF6, in turn, is transported from the ER to the Golgi apparatus, where it undergoes proteolytic activation before translocating to the nucleus to induce the expression of chaperone proteins, folding enzymes, and quality control machinery, thereby limiting the accumulation of misfolded proteins and preserving secretory capacity [10,12]. Under physiological conditions, this adaptive UPR maintains proteostasis and supports the sustainability of decidualization [9,12]. However, under conditions of chronic ER stress, the PERK–ATF4 axis promotes increased expression of C/EBP homologous protein (CHOP), which suppresses the anti-apoptotic protein Bcl-2 and activates pro-apoptotic mediators such as Bax and Bak, ultimately inducing mitochondrial apoptosis [9,16]. Concurrently, prolonged activation of IRE1α engages tumor necrosis factor receptor-associated factor 2 (TRAF2), leading to the activation of c-Jun N-terminal kinase (JNK) signaling and increased production of pro-inflammatory cytokines via nuclear factor kappa B (NF-κB), thereby shifting the decidual microenvironment toward a pro-inflammatory state [10,16]. The interaction between ER stress and mitochondria further exacerbates metabolic imbalance through increased production of ROS and disruption of calcium homeostasis. These processes are closely interconnected with AMPK signaling, collectively influencing cellular energy status [21,30]. The major ER stress–UPR pathways involved in decidualization and their downstream effects are illustrated in Figure 1.

In summary, early and tightly regulated activation of the UPR functions as a “physiological buffering mechanism” in decidual stromal cells, preserving proteostasis and supporting secretory capacity. In contrast, prolonged and uncontrolled ER stress drives a maladaptive trajectory characterized by apoptosis, inflammation, and metabolic dysfunction, ultimately compromising the integrity of decidualization [9,16]. This dual nature underscores that ER stress is not merely a cellular stress response but represents a critical decision-making node in decidual biology.

## 4. Adaptive–Pathological Transition of ER Stress

ER stress functions as a dynamic threshold system in decidual stromal cells, with its outcome determined by the duration, intensity, and cellular context of the stress signal [9,12,16]. Low-level and transient ER stress induces an adaptive phase mediated by the UPR. During this phase, the PERK–eIF2α axis attenuates translational load, while the IRE1α–XBP1 and ATF6 pathways enhance protein-folding capacity and secretory function, thereby supporting the sustainability of decidualization [9,10]. In contrast, when ER stress is prolonged or intensified, the system surpasses a critical threshold and transitions into a maladaptive phase. This shift is characterized by increased activation of C/EBP homologous protein (CHOP) via the PERK–ATF4 axis, suppression of anti-apoptotic signaling, and induction of mitochondrial apoptosis [9,16]. Concurrently, sustained activation of IRE1α engages the c-Jun N-terminal kinase (JNK) and nuclear factor kappa B (NF-κB) pathways, amplifying the inflammatory response and exacerbating cellular injury [10,16]. This transition extends beyond the cellular level, directly impacting immune and metabolic homeostasis within the decidual microenvironment. Chronic ER stress is associated with increased production of ROS, disruption of calcium homeostasis, and mitochondrial dysfunction, all of which impair cellular energy balance and accelerate the progression of UPR toward its maladaptive phase [21,30]. These alterations culminate in enhanced pro-inflammatory cytokine production and suppression of cell survival mechanisms [16]. Within this framework, ER stress can be conceptualized as a “molecular decision switch” in decidual biology, governing the transition between adaptation and injury. While it operates as a protective mechanism that preserves proteostasis and secretory capacity under physiological conditions, exceeding these limits triggers a pathological cascade characterized by inflammation, apoptosis, and functional impairment [9,12,16].

## 5. Immune–Endoplasmic Reticulum Stress Interactions

**Figure 2:**
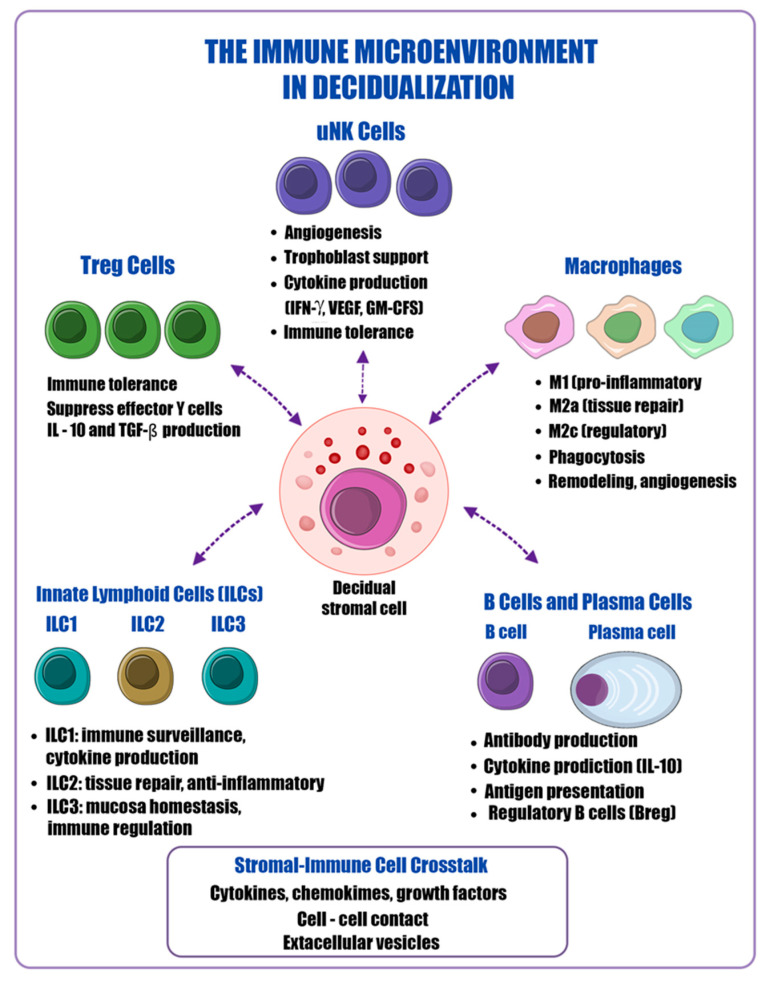
Immune microenvironment in decidualization. Schematic overview of immune cell populations interacting with decidual stromal cells during decidualization. uNK cells, macrophages, Treg cells, ILC1–3, and B/plasma cells contribute to angiogenesis, tissue remodeling, immune tolerance, and cytokine production. Crosstalk between stromal and immune cells is mediated by cytokines, chemokines, growth factors, direct cell–cell contact, and extracellular vesicles, collectively supporting a regulated decidual immune environment.

The decidual immune microenvironment comprises an organized regulatory network of uNK cells, regulatory T (Treg) cells, macrophage subsets, innate lymphoid cells (ILCs), and B cells [5,14,29]. Within this network, ER stress and the associated UPR function as central regulators that determine the balance between inflammation and immune tolerance [10,13]. The major immune cell populations and their interactions with decidual stromal cells during decidualization are illustrated in Figure 2.

Macrophages in the decidua exhibit tissue-specific functional phenotypes that extend beyond the classical M1/M2 paradigm. CD206^+^ and CD163^+^ immunoregulatory macrophages support a tolerogenic microenvironment through the secretion of transforming growth factor beta (TGF-β) and interleukin-10 (IL-10). However, increased ER stress has been associated with a shift toward pro-inflammatory phenotypes via activation of the PERK–CHOP and IRE1α–JNK axes [11,16]. This phenotypic transition, in concert with stromal cell-derived chemokines, may contribute to amplification of the inflammatory response [5,10]. In the context of innate lymphoid cells, not only ILC3 but also ILC1 and ILC2 subsets play roles in decidual physiology. ILC1 cells can limit trophoblast invasion through interferon-gamma (IFN-γ) production, whereas ILC2 cells support tissue repair via interleukin-5 (IL-5) and interleukin-13 (IL-13) [29]. Chronic ER stress may alter cytokine profiles, particularly through IRE1α–XBP1 signaling, thereby influencing the functional balance of these cell populations [10]. B cells and plasma cells represent additional key components of decidual immune tolerance. Regulatory B cells (Bregs) promote Treg stability through IL-10 production, while plasma cells contribute to the suppression of immune responses against trophoblast antigens [13]. ER stress is known to regulate plasma cell differentiation through XBP1 signaling; physiological UPR activation supports antibody secretion, whereas excessive stress may lead to the accumulation of misfolded immunoglobulins and apoptosis, ultimately impairing humoral tolerance [10].

Decidual stromal cells themselves act as active regulators of the immune microenvironment. Through mediators such as prostaglandin E2 (PGE2), indoleamine 2,3-dioxygenase (IDO), TGF-β, and IL-15, they orchestrate uNK cell function, Treg differentiation, and macrophage polarization [5,14]. Increased ER stress has been reported to reprogram these immunoregulatory functions by altering both secretory capacity and cytokine profiles [9,12,16]. Activating transcription factor 6 (ATF6), by regulating ER chaperone responses and secretory capacity, may influence the production of immunomodulatory cytokines by decidual stromal cells and thereby contribute to immune tolerance processes [10,12]. Conversely, the impairment of ATF6 function may be associated with secretory insufficiency and loss of immune tolerance [9,12]. Overall, ER stress emerges as a higher-order regulatory axis that reshapes interactions between immune and stromal cells. While maintenance of this axis is essential for immune tolerance and successful implantation, its chronic activation may disrupt the inflammation–tolerance balance, thereby predisposing to pathological outcomes [10,13,16].

## 6. Metabolic–Endoplasmic Reticulum Stress Interactions

Endometrial decidualization is not solely a hormonally driven differentiation process but also a metabolic adaptation program that requires the reorganization of cellular energy homeostasis [17,18,28]. In this context, metabolic reprogramming represents a critical determinant enabling stromal cells to meet the increased demands of protein synthesis and secretory capacity [17,18]. AMPK, a key cellular energy sensor, is activated under conditions of energy imbalance and occupies a central role in this process [19,31]. Reciprocal interactions between ER stress and metabolic reprogramming, particularly mediated through AMPK signaling, have been described, suggesting a coordinated regulation of cellular energy balance and proteostasis [19,20,21]. Activation of AMPK suppresses the mechanistic target of rapamycin (mTOR) pathway, thereby limiting protein synthesis, reducing the translational burden on the ER, and contributing to the maintenance of proteostasis [21,32]. In addition, AMPK promotes autophagy, facilitating the clearance of damaged proteins and dysfunctional organelles [32]. When this metabolic balance is disrupted, particularly in the setting of mitochondrial dysfunction, intracellular redox homeostasis becomes compromised [18]. Increased production of ROS may interfere with protein-folding processes, contributing to the accumulation of misfolded proteins within the ER and triggering ER stress responses [21]. At this stage, UPR activation initially serves as an adaptive mechanism aimed at enhancing protein-folding capacity and preserving cellular homeostasis; however, under chronic conditions, it may shift toward a maladaptive profile associated with pro-apoptotic and pro-inflammatory signaling pathways [9,10,16]. A critical determinant of this transition is the bidirectional crosstalk between the ER and mitochondria. Elevated ROS levels can exacerbate ER stress, while ER-derived calcium release may disrupt mitochondrial function and further amplify oxidative stress-related alterations [30]. This reciprocal interaction establishes a positive feedback loop, contributing to progressive metabolic imbalance and increased cellular stress burden [21,30].

In summary, while the AMPK–ER stress interaction initially exhibits adaptive features, conditions of chronic energy insufficiency and oxidative stress may drive cellular responses associated with apoptosis, inflammation, and functional impairment [16,21]. Accordingly, the AMPK–mitochondria–ER stress axis can be considered a key regulatory network influencing the balance between cellular adaptation and dysfunction during decidualization [19,30].

## 7. Integration of the ER Stress–Immune–Metabolic Axis

**Figure 3:**
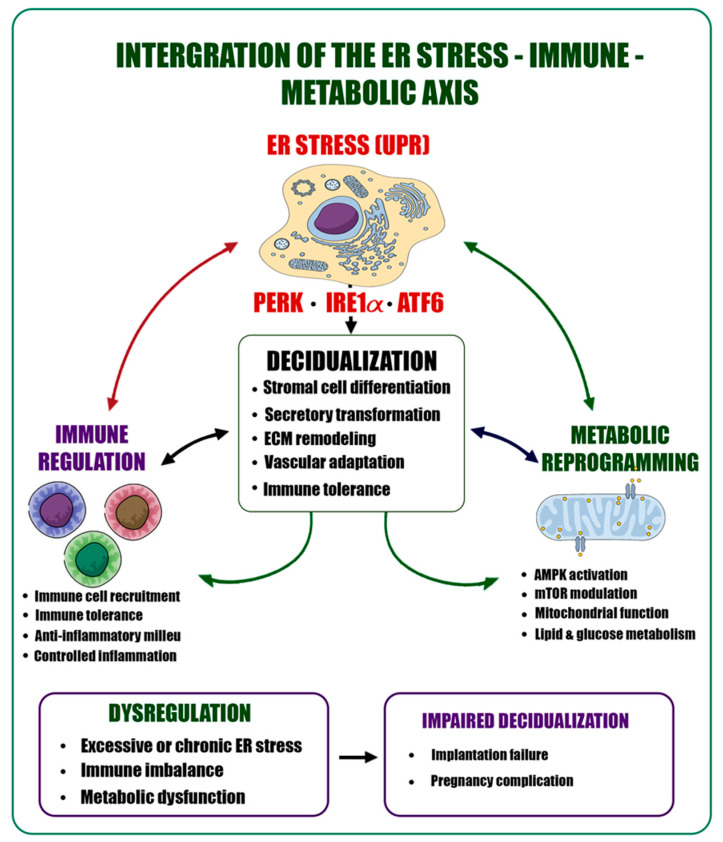
Integration of the ER stress–immune–metabolic axis in decidualization. Schematic representation of the interplay between ER stress (UPR; PERK, IRE1α, ATF6), immune regulation, and metabolic reprogramming during decidualization. Coordinated signaling supports stromal cell differentiation, secretory transformation, ECM remodeling, vascular adaptation, and immune tolerance. Dysregulation of this axis, including excessive ER stress, immune imbalance, or metabolic dysfunction, is associated with impaired decidualization, implantation failure, and pregnancy complications.

Decidualization represents a multilayered biological organization in which cellular stress responses, immune regulation, and metabolic adaptation are tightly integrated, rather than a unidimensional differentiation process [3,4,5,17,18]. At the core of this integrative network, ER stress functions as a strategic regulatory node that extends beyond proteostasis to orchestrate immune microenvironmental dynamics and metabolic reprogramming [10,13]. The UPR, activated in response to ER stress, initially supports cellular adaptation while simultaneously modulating immune and metabolic processes [9,10,12]. The integration of ER stress signaling with immune regulation and metabolic reprogramming during decidualization is illustrated in Figure 3. Low-level ER stress has been associated with the establishment of a tolerogenic microenvironment through its effects on macrophage polarization, uNK cell activity, and T cell balance. In contrast, chronic ER stress is linked to increased production of pro-inflammatory cytokines and activation of inflammatory signaling pathways, contributing to immune imbalance [13,14,16]. In this context, ER stress functions as a higher-order regulator that reprograms immune cell behavior [10,13].

From a metabolic perspective, ER stress acts as an active determinant of metabolic reprogramming. AMPK signaling operates in close crosstalk with the UPR, coordinating protein synthesis, lipid metabolism, and autophagy [21,32]. Increased energetic demand is associated with AMPK activation, which promotes catabolic processes and redistribution of cellular resources [19,31]. As metabolic stress intensifies, mitochondrial dysfunction and ROS production increase, further exacerbating ER stress and establishing a pathological feedback loop [21,30]. A defining feature of this axis is its non-linear, bidirectional network architecture, characterized by dynamic feedback mechanisms. While ER stress regulates immune and metabolic processes, inflammatory cytokines and metabolic imbalance, in turn, enhance UPR activation, reinforcing the system through reciprocal interactions [10,16]. Thus, ER stress serves as an integrative platform linking immune and metabolic pathways. Within this framework, decidualization failure may be viewed not as the sum of independent events but as the outcome of a sequential mechanistic cascade centered on ER stress, in which immune activation and metabolic disruption mutually reinforce each other [16]. Chronic UPR activation may impair stromal cell function through increased inflammation, loss of immune tolerance, and energy imbalance, ultimately leading to reduced secretory capacity and compromised endometrial receptivity [9,12,16,30].

In conclusion, the ER stress–immune–metabolic axis can be conceptualized as a central regulatory system determining the fate of decidualization. The balance of this axis is a key determinant of implantation success, and its targeted modulation holds considerable translational potential, particularly in the context of recurrent implantation failure and pregnancy loss [17].

## 8. Bidirectional Crosstalk

Within the decidual microenvironment, ER stress functions not as a passive byproduct but as a central regulator that actively reshapes immune and metabolic processes [10,13]. The UPR, triggered by ER stress, establishes a dynamic control platform that coordinates bidirectional interactions between immune and metabolic axes [9,10,12]. At the immune level, UPR signaling pathways influence macrophage polarization, T cell subset balance, and uNK cell function through the modulation of cytokine and chemokine production [5,10,14]. Activation of the IRE1α–XBP1 pathway has been associated with the production of secretory and immunomodulatory proteins, whereas the PERK–CHOP axis is linked to enhanced inflammatory responses [10,16]. Conversely, inflammatory cytokines such as tumor necrosis factor alpha (TNF-α) and interleukin-1 beta (IL-1β) can exacerbate ER stress, thereby triggering UPR activation and establishing a reciprocal amplification loop between the ER and the immune system [16]. A similar bidirectional relationship is observed at the metabolic level. The activation of AMPK under conditions of energy deprivation suppresses protein synthesis, reduces the burden on the ER, and supports adaptive UPR signaling [21,32]. In contrast, mitochondrial dysfunction leading to increased production of ROS may disrupt protein-folding processes and further intensify ER stress [21]. The UPR, in turn, regulates key metabolic processes, including lipid biosynthesis, glucose metabolism, and autophagy, thereby contributing to metabolic reprogramming [10,21].

Collectively, these reciprocal interactions demonstrate that the ER stress–immune–metabolic axis operates not as a linear pathway but as a dynamic, context-dependent network characterized by feedback regulation [10,16]. Accordingly, this axis can be regarded as a fundamental regulatory system governing the success of decidualization [10,13].

## 9. Pathological Conditions: Disruption of the Endoplasmic Reticulum Stress–Immune–Metabolic Axis

**Figure 4:**
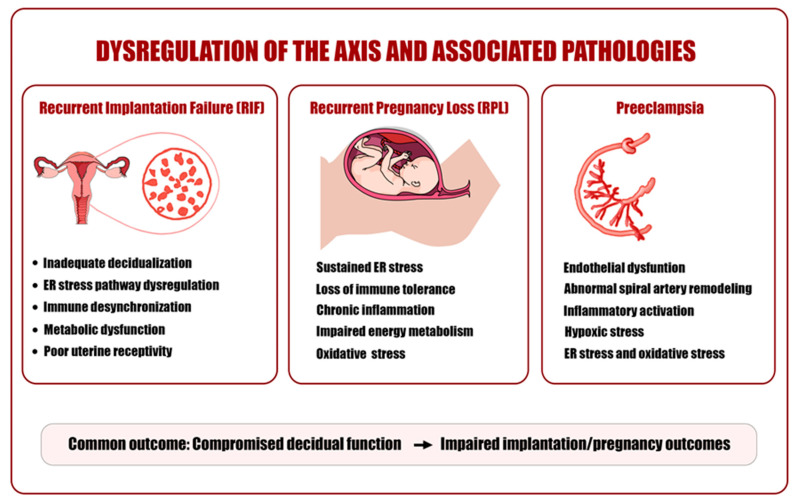
Dysregulation of the ER stress–immune–metabolic axis and associated pathologies. Overview of conditions associated with axis dysregulation, including recurrent implantation failure (RIF), recurrent pregnancy loss (RPL), and preeclampsia. These states are characterized by inadequate decidualization, persistent ER stress, immune imbalance, inflammation, and metabolic dysfunction. Collectively, these alterations are associated with compromised decidual function and impaired implantation and pregnancy outcomes.

Pathological conditions such as recurrent implantation failure, pregnancy loss, and preeclampsia are associated with disruption of the ER stress–immune–metabolic axis and may be interpreted not as isolated events but as components of a sequential mechanistic cascade centered on ER stress [16]. This cascade is initiated by increased protein synthesis and metabolic load, which activate an adaptive UPR [9,10]. With chronic stress, this response transitions to a maladaptive phase, leading to secretory dysfunction in stromal cells [9,12,16]. At this stage, decreased expression of receptivity markers such as prolactin, IGFBP-1, leukemia inhibitory factor (LIF), and integrin αvβ3 has been associated with impaired epithelial–stromal interactions [23,26]. Concurrently, the immune microenvironment undergoes reprogramming; increased inflammatory cytokine production weakens the tolerogenic profile, with shifts toward pro-inflammatory macrophage phenotypes, disruption of the Treg/Th17 balance, and altered uNK cell function [5,10,14]. At the metabolic level, mitochondrial dysfunction and increased ROS production further exacerbate ER stress, forming a positive feedback loop [21,30]. This condition is associated with energy insufficiency, increased cellular stress burden, apoptosis, and reduced functional capacity in stromal cells [16,21]. Overall, this multilayered disruption of the ER stress–immune–metabolic axis is linked to impaired decidualization capacity and loss of endometrial receptivity [17]. The pathological consequences associated with dysregulation of the ER stress–immune–metabolic axis are illustrated in Figure 4. Notably, this process is not linear but is characterized by interconnected feedback loops in which ER stress, immune activation, and metabolic dysfunction mutually reinforce one another [10,16]. Increased inducible nitric oxide synthase (iNOS) expression and inflammatory activation may represent clinical manifestations of ER stress–immune interactions, while impaired pinopode development and altered epithelial surface morphology may reflect structural indicators of defective decidualization [28,33].

### 9.1. Recurrent Implantation Failure (RIF)

RIF represents a complex pathophysiological condition characterized by impaired endometrial receptivity and insufficient decidualization [17,28]. Increased ER stress has been associated with alterations in stromal cell secretory capacity and protein-folding processes [9,12,16]. Although UPR initially plays an adaptive role, its chronic activation may contribute to apoptosis and activation of inflammatory signaling pathways [16]. This is reflected in altered expression of receptivity markers such as LIF and integrin αvβ3, along with disruption of epithelial–stromal interactions [23,26]. At the immune level, ER stress is associated with imbalances among uNK cells, macrophages, and T cell subsets, accompanied by increased pro-inflammatory cytokine production and attenuation of the tolerogenic profile [5,10,14]. Metabolically, mitochondrial dysfunction and elevated ROS production further intensify ER stress, while energy imbalance limits decidualization capacity [21,30]. Collectively, RIF is characterized by multifaceted dysfunction of the ER stress–immune–metabolic axis, with disturbances in this axis representing key determinants of implantation failure [17].

### 9.2. Recurrent Pregnancy Loss (RPL)

RPL is characterized by the failure to maintain maternal–fetal immune tolerance following implantation, with chronic ER stress proposed as a contributing factor [17]. ER stress-associated immune dysregulation may manifest as reduced Treg cell populations and increased Th1/Th17 responses, resulting in a pro-inflammatory profile that compromises tolerance to the semi-allogeneic embryo [10,13]. Additionally, ER stress may promote macrophage polarization toward pro-inflammatory phenotypes, further enhancing cytokine production [10,34]. At the metabolic level, increased oxidative stress and ROS production may reinforce ER stress responses, forming a pathological feedback loop [21,30]. Disruption of energy homeostasis and mitochondrial insufficiency are associated with impaired decidual cell function and reduced pregnancy sustainability [21]. Thus, RPL can be conceptualized as a multilayered pathophysiological condition in which ER stress-mediated loss of immune tolerance and metabolic dysfunction converge [17].

### 9.3. Preeclampsia

Preeclampsia is a severe obstetric syndrome that emerges in later stages of pregnancy and is characterized by abnormal placental development [34]. Central to its pathogenesis is inadequate trophoblast invasion and impaired physiological remodeling of spiral arteries [34,35]. Increased ER stress and UPR dysregulation have been implicated in altered trophoblast proliferation, migration, and invasion capacities [16,36]. Chronic ER stress may also contribute to increased apoptosis in trophoblast cells and dysregulation of angiogenic factors, ultimately leading to endothelial dysfunction [33]. At the metabolic level, elevated oxidative stress and mitochondrial dysfunction are associated with damage to both placental and maternal vascular structures [21,30]. From an immunological perspective, increased inflammatory cytokine levels and disruption of the tolerogenic profile contribute to systemic inflammation and may exacerbate disease severity [16]. In summary, preeclampsia represents a complex syndrome situated at the intersection of ER stress–UPR dysregulation, immune activation, and metabolic imbalance, with disturbances in this axis affecting both local placental development and systemic maternal responses [33,34].

## 10. Therapeutic Perspectives

**Figure 5:**
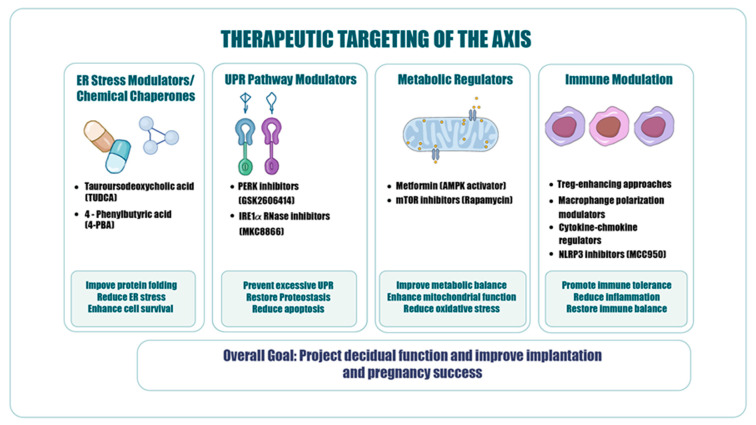
Therapeutic targeting of the ER stress–immune–metabolic axis. Overview of potential therapeutic strategies targeting ER stress, UPR pathways, metabolic regulation, and immune modulation. Approaches including chemical chaperones, pathway-specific inhibitors, metabolic regulators, and immunomodulatory interventions are associated with restoration of cellular homeostasis, reductions in inflammation and apoptosis, and improved decidual function, with the overall aim of enhancing implantation and pregnancy outcomes.

Modulation of the ER stress–immune–metabolic axis, which critically determines decidualization and implantation success, has emerged as a priority area in contemporary translational research [16]. Targeting this axis requires multilayered strategies capable of simultaneously regulating proteostasis, inflammation, and energy homeostasis [10,13,21]. Potential therapeutic approaches targeting the ER stress–immune–metabolic axis are illustrated in Figure 5.

Chemical chaperones represent a key therapeutic class by facilitating proper protein folding and reducing the accumulation of misfolded proteins within the ER, thereby supporting proteostasis. Tauroursodeoxycholic acid and 4-phenylbutyric acid are among the most extensively characterized agents in this context [29,37]. These compounds have been reported to attenuate excessive activation of ER stress sensors, limit CHOP-mediated apoptosis and inflammatory responses, and exert protective effects on mitochondrial function and oxidative stress [16,29]. Selective modulation of the principal UPR branches provides opportunities for targeted therapeutic interventions. Inhibitors of IRE1α RNase activity (e.g., MKC8866) can suppress XBP1 splicing, thereby limiting processes associated with excessive adaptive and pro-inflammatory responses [38]. Similarly, PERK inhibitors (e.g., GSK2606414) may modulate mechanisms related to chronic ER stress-induced apoptosis through effects on eIF2α phosphorylation [39]. However, given their potential to also suppress physiologically adaptive UPR signaling, careful consideration of dosing and timing is essential [9,10]. Targeting the metabolic axis, particularly through AMPK activation, represents another central strategy. Metformin activates AMPK, suppresses mechanistic target of rapamycin (mTOR) signaling, reduces the protein synthesis burden, and enhances autophagy [21,40]. These effects are associated with reduced ER load, improved mitochondrial function, and modulation of inflammatory cytokine production, potentially contributing to the maintenance of a tolerogenic microenvironment [21,40]. Inhibition of the NLRP3 inflammasome constitutes a critical approach for targeting the immune axis. MCC950 suppresses NLRP3 activation, reduces interleukin-1 beta (IL-1β) and interleukin-18 (IL-18) production, and limits processes associated with chronic inflammation [33]. This effect may be linked to the disruption of the ER stress–inflammation feedback loop [16]. Collectively, these agents aim to modulate pathological feedback circuits within the ER stress–immune–metabolic axis and restore the system toward an adaptive equilibrium. Nevertheless, the highly integrated nature of this axis suggests that single-agent interventions may be insufficient. Future therapeutic strategies should therefore prioritize rational combination approaches incorporating chemical chaperones, UPR modulators, metabolic regulators, and anti-inflammatory agents, with an emphasis on personalized and temporally optimized treatment protocols [10,21].

## 11. Interspecies Differences, Limitations, and Future Perspectives

Significant differences exist between human and animal models in terms of decidualization, immune responses, and metabolic regulation, necessitating careful interpretation of translational findings [2,3]. In humans, decidualization occurs spontaneously under the influence of progesterone and cyclic adenosine monophosphate (cAMP), whereas in rodents, it is primarily triggered by implantation, leading to important differences in the timing of early molecular events [2]. From an immunological perspective, the human decidua exhibits a highly tolerogenic profile, while animal models may differ in cellular composition and cytokine landscapes [6,7]. Consistent with both mechanistic studies and clinical observations, the effects of ER stress on immune cell function may vary across species [10,11,13]. At the metabolic level, interspecies differences in hormonal cyclicity, mitochondrial activity, and AMPK-mediated energy regulation are also evident [19,31]. Furthermore, the deeper trophoblast invasion observed in humans limits the ability of animal models to fully recapitulate pathologies such as preeclampsia [34]. Beyond these translational limitations, the current literature reveals important conceptual and methodological gaps. The adaptive and pathological roles of ER stress have largely been described at a descriptive level, while the threshold mechanisms governing the transition between these states remain insufficiently defined [9,12,16]. Although the contribution of ER stress to decidualization failure is commonly attributed to apoptosis, inflammation, and metabolic disruption, the integration of these processes into a cohesive causal framework linking stromal cell dysfunction and alterations in receptivity markers has not been fully elucidated [16]. In the context of metabolic reprogramming, bidirectional interactions between AMPK, mitochondrial function, and ER stress have been identified; however, whether the UPR actively orchestrates metabolic remodeling or primarily responds to it remains unresolved [21,30]. From an immunological standpoint, although ER stress has been shown to influence macrophage, T cell, and B cell functions, the cell type-specific temporal and spatial dynamics of these effects remain largely unknown [10,13,34]. These limitations largely stem from the predominantly static and correlative nature of existing data, underscoring the need for functional, time-resolved, and cell-specific approaches to establish causal relationships [16]. Accordingly, future studies should prioritize the use of single-cell omics technologies, spatial transcriptomic analyses, and functional in vivo models to elucidate the dynamic regulation of the ER stress–immune–metabolic axis [7,8]. Such approaches will not only deepen our understanding of decidual biology but also provide a robust foundation for the development of targeted and personalized therapeutic strategies [8].

## 12. Conclusions

The human endometrium should be considered not a passive target tissue but a central regulatory system that coordinates immune tolerance and metabolic adaptation through ER stress and the UPR. During decidualization, ER stress extends beyond the maintenance of proteostasis to regulate immune cell function, cytokine production, and the balance between inflammation and tolerance while simultaneously engaging in bidirectional interactions with energy homeostasis, mitochondrial function, and redox balance at the metabolic level. Within this framework, the ER stress–immune–metabolic axis represents a critical threshold mechanism governing the transition between cellular adaptation and pathological transformation. Despite these advances, significant knowledge gaps remain. The temporal dynamics of UPR activation, its cell type-specific effects on immune cell subsets, and its causal relationships with metabolic pathways are not yet fully elucidated. Moreover, the molecular boundaries distinguishing physiological from pathological ER stress responses, and how these thresholds may be modulated in a clinical context, remain unclear. Interspecies differences and the inherent limitations of current experimental models further constrain translational interpretation. Future investigations should therefore prioritize the application of single-cell omics technologies, spatial transcriptomics, and functional in vivo models to delineate the cell type-specific and time-dependent regulation of the ER stress–immune–metabolic axis. Integrative therapeutic strategies that combine ER stress- and UPR-targeted pharmacological modulation with interventions addressing immune and metabolic pathways hold substantial promise for the development of next-generation, personalized treatments in reproductive medicine. In conclusion, ER stress represents not merely a cellular stress response but an integrated control platform that determines the trajectory of decidual biology. Targeting this axis is likely to remain a priority research area, with significant implications for both advancing fundamental scientific understanding and improving clinical outcomes.

## Data Availability

Data sharing is not applicable to this article as no new data were created or analyzed in this study.
